# A low-volume polyethylene glycol solution was associated with an increased suboptimal bowel preparation rate but had similar recommendations for an early repeat colonoscopy, procedure times, and adenoma detection rates

**DOI:** 10.1371/journal.pone.0176265

**Published:** 2017-04-27

**Authors:** Sam C. Hankins, Bryan B. Brimhall, Vineel Kankanala, Gregory L. Austin

**Affiliations:** 1 University of Texas Southwestern Medical Center, Dallas, Texas, United States of America; 2 Division of Gastroenterology and Hepatology, University of Colorado Anschutz Medical Campus, Aurora, Colorado, United States of America; 3 Division of Gastroenterology and Hepatology, University of Illinois at Chicago, Chicago, Illinois, United States of America; University Hospital Llandough, UNITED KINGDOM

## Abstract

**Background/Aims:**

Low-volume polyethylene glycol (PEG) bowel preparations are better tolerated by patients than high-volume preparations and may achieve similar preparation quality. However, there is little data comparing their effects on a recommendation for an early repeat colonoscopy (because of a suboptimal preparation), procedure times, adenoma detection rate (ADR), and advanced adenoma detection rate (AADR).

**Methods:**

This is a retrospective cohort study of outpatient colonoscopies performed during a one-year period at a single academic medical center in which low-volume MoviPrep^®^ (n = 1841) or high-volume Colyte^®^ (n = 1337) was used. All preparations were split-dosed. Appropriate covariates were included in regression models assessing suboptimal preparation quality (fair, poor, or inadequate), procedure times, recommendation for an early repeat colonoscopy, ADR, and AADR.

**Results:**

MoviPrep^®^ was associated with an increase in having a suboptimal bowel preparation (OR 1.36; 95% CI: 1.06–1.76), but it was not associated with differences in insertion (p = 0.43), withdrawal (p = 0.22), or total procedure times (p = 0.10). The adjusted percentage with a suboptimal preparation was 11.7% for patients using MoviPrep^®^ and 8.8% for patients using Colyte^®^. MoviPrep^®^ was not associated with a significant difference in overall ADR (OR 0.93; 95% CI: 0.78–1.11), AADR (OR 1.18; 95% CI: 0.87–1.62), or recommendation for early repeat colonoscopy (OR 1.16; 95% CI: 0.72–1.88).

**Conclusions:**

MoviPrep^®^ was associated with a small absolute increase in having a suboptimal preparation, but did not affect recommendations for an early repeat colonoscopy, procedure times, or adenoma detection rates. Mechanisms to reduce financial barriers limiting low-volume preparations should be considered because of their favorable tolerability profile.

## Introduction

Colorectal cancer (CRC) is the second overall cause of cancer-related mortality in the United States [1]. Colonoscopy has become the most commonly utilized screening method to detect and remove suspicious colonic polyps with the intent of preventing CRC [2]. Screening for CRC with colonoscopy has been shown to be an effective, yet imperfect, mode of finding and removing precancerous polyps (adenomas). Studies suggest late-stage CRC is discovered at higher rates in those with low rates of having undergone a screening colonoscopy [3]. Critical to a high-quality screening colonoscopy is that the patient achieve a high-quality bowel preparation, generally one supplemented with polyethylene glycol (PEG), allowing the mucosal surfaces of the entire colon to be easily visualized by the endoscopist. A suboptimal bowel preparation may allow a neoplastic lesion to go undetected. An ideal bowel preparation agent would achieve a high rate of a high-quality bowel preparation, be well-tolerated by a high proportion of patients, and be inexpensive [4].

Several bowel preparation agents for colonoscopy are available. Currently, the primary choices for bowel cleansing are either high-volume (4L) PEG solutions or low-volume (approximately 2L) PEG solutions [5]. The high-volume solutions include Colyte^®^, Golytely^®^, and Nulytely^®^. Low-volume solutions include MoviPrep^®^ and Suprep^®^. MoviPrep^®^ is a PEG solution that also contains ascorbic acid, which improves palatability. All of these aforementioned products contain PEG polymers, making them iso-osmotic. This prevents rapid shifts in fluid and electrolytes in the gastrointestinal tract. Therefore, they are a safe choice for prepping the bowel.

Recent studies have shown varying degrees of efficacy between the high and low-volume regimens, usually revealing an insignificant difference in expert-rated opinions on the quality of the bowel preparation [6–8]. Preparations are also more effective when given in a split-dose schedule as compared to a non-split-dosage schedule [9]. Low-volume solutions, however, consistently rank better on patient-completed questionnaires with respect to tolerability, taste, and adverse effects like bloating, nausea, vomiting, and general discomfort [6, 10, 11]. The higher rate of side effects associated with high-volume preparations seems to be directly linked with higher degrees of incomplete adherence (to the bowel preparation instruction) [12]. The importance of this issue is related to the fact that a suboptimal bowel preparation stemming from incomplete adherence could lead to missed neoplastic lesions. Additionally, this could result in the need to repeat the colonoscopy sooner than would otherwise be clinically indicated, leading to increased health care costs. Low-volume preparations, like MoviPrep^®^, may have better adherence, but are not covered under several common Medicaid or Medicare policies. If the lack of adherence associated with a high-volume preparation were to compromise the quality of the bowel preparation, it may be more favorable to extend coverage for a low-volume bowel preparation regimen for these patients.

There is relatively little data beyond preparation quality and tolerability comparing high-volume to low-volume bowel preparations for important colonoscopy outcomes. The purpose of our study was to analyze novel colonoscopy outcomes based on whether patients used a high-volume bowel preparation compared to a low-volume bowel preparation. Specifically, the objectives of this study were to compare Colyte^®^ to MoviPrep^®^ with respect to suboptimal preparation quality, recommendation for an early repeat colonoscopy secondary to a suboptimal bowel preparation, insertion time, withdrawal time, total procedural time, adenoma detection rate (ADR), and advanced adenoma detection rate (AADR).

## Methods

This is a cohort study of all outpatient colonoscopies performed at the University of Colorado Hospital from October 2011 through October 2012. The methods for this study are similar to those that have been previously published by our group [13]. This study was approved by the Colorado Multiple Institutional Review Board.

### Study population

Patients who underwent an outpatient colonoscopy during the study period and consumed either Colyte^®^ or MoviPrep^®^ prior to the procedure were included. Each bowel preparation solution was given in a split-dose schedule (half of the preparation the night before the procedure and the other half the morning of the procedure), as this method has proven to be more efficacious with respect to bowel preparation quality [9]. Only 22 procedures were excluded because an alternate preparation was utilized. UCH’s electronic medical record (Epic; Verona, WI) and the endoscopy reporting database (Provation^®^; Minneapolis, MN) were used to extract covariate and outcome data pertinent to this study. Patients with a history of inflammatory bowel disease (n = 127) and a personal history of CRC (n = 74) were excluded. Patients with an indication for a fecal transplant secondary to clostridium difficile colitis were also excluded (n = 4). In total, 1841 subjects used MoviPrep^®^, while 1337 used Colyte^®^.

### Predictor variables and outcomes

Covariates that were extracted were age, gender, indication for colonoscopy (diagnostic versus screening/surveillance), fellow involvement, need for an interpreter, having a chronic pain diagnosis, outpatient use of opiate medications, the individual attending endoscopist, and the insurance provider of the patient. Additionally, we extracted the total number of polyps and the size of the largest polyp for each patient as these have a significant effect on withdrawal time and total procedure time. Because of differences in procedure time outcomes (and the detection of adenomas) between endoscopists, appropriate dummy variables for each endoscopist (n = 8) were used during all multivariate analyses. Insurance status was divided into the following five categories and included as covariates in the multivariable regression analyses for all outcomes: (1) commercial insurance (e.g., Cigna, United, etc.; n = 1343); 2) Medicare ≥ 65y (n = 835); 3) Medicare < 65y (n = 182); 4) Tricare (government insurance for military personnel and their dependents; n = 633), and 5) Medicaid (n = 185).

The outcomes that were investigated were bowel preparation quality, insertion time, withdrawal time, total procedure time, the overall adenoma detection rate (ADR), the overall advanced adenoma detection rate (AADR), and the recommendation for an early repeat colonoscopy secondary to a suboptimal bowel preparation. The quality of the bowel preparation was rated by the individual endoscopist at the time of the procedure. The rating scale for each individual’s preparation quality was based upon the modified Aronchick scale. This scale uses the following criteria: *Poor*/*Inadequate*–poor preparation quality, exam still completed, feces and/or turbid fluid make preparation unreliable and less than 90% of the mucosa is visualized; *Fair*–moderate amount of stool that may be adequately cleared via suctioning to permit adequate evaluation, over 90% of the mucosa can be visualized; *Good*–some turbid fluid without feces, no interference with exam, more than 90% of mucosa visualized; *Excellent*–small amount of clear liquid with over 95% of the mucosa visualized [14]. Preparation quality was dichotomized into optimal (good or excellent) and suboptimal (fair, poor, or inadequate). Total procedure time was calculated from the time stamps in Provation^®^ that identify “Scope In” as the start of the procedure and “Scope Out” as the completion of the procedure. Insertion time was calculated from the time stamps that identify “Scope In” and “Cecum Reached”. Withdrawal time was calculated from the time stamps that identify “Cecum Reached” and “Scope Out”. The ADR was calculated as the percentage of patients in each group who had at least one adenoma or sessile serrated polyp/adenoma. The AADR was calculated as the percentage of patients in each group who had at least one advanced adenoma on the basis of size (any adenoma or sessile serrated polyp ≥ 10mm) or histology (adenomas containing villous histology [or high-grade dysplasia] regardless of size and sessile serrate polyps with dysplasia). For patients with a suboptimal preparation, an early repeat colonoscopy was defined when the interval that was recommended was clearly not indicated based on the findings of the colonoscopy, a patient’s family history, or a patient’s prior history of adenomatous polyps or cancer.

### Statistical analysis

All data were entered into and analyzed using STATA 10.0 statistical software (StataCorp, College Station, Texas). Demographic and baseline characteristics for the two groups (Colyte^®^ and MoviPrep^®^) were compared using the t-test and the chi-square test. The following covariates were included in the multivariable linear regression models for insertion, withdrawal and total procedure time: age, gender, indication for colonoscopy (diagnostic versus screening/surveillance), fellow involvement, need for an interpreter, a chronic pain diagnosis, outpatient use of opiate medications, total number of polyps resected, size of the largest polyp resected, the individual attending endoscopist, and the insurance provider of the patient. The following covariates were included in the multivariable logistic regression models for suboptimal preparation quality, recommendation for an early repeat colonoscopy, ADR, and AADR: age, gender, indication for colonoscopy (diagnostic versus screening/surveillance), fellow involvement, need for an interpreter, a chronic pain diagnosis, outpatient use of opiate medications, the individual attending endoscopist, and the insurance provider of the patient. Because there were differences in the covariates between the groups, we calculated adjusted percentages (for the outcomes of suboptimal preparation quality, recommendation for an early repeat colonoscopy, ADR, and AADR) and adjusted means (for procedure time outcomes) using the “predxcat” command in STATA.

## Results

A total of 3,178 colonoscopies meeting the inclusion criteria were used for this study. There were some differences between the two groups (Table 1). There was a significant difference in the percentage of colonoscopies completed as diagnostic procedures between the two bowel preparations, with 25.3% of MoviPrep^®^ patients and 21.5% of Colyte^®^ patients undergoing colonoscopies for diagnostic indications (p = 0.02). The percentage of cases in which a fellow was involved in the colonoscopy was also statistically significant with a fellow being involved in 22.7% and 12.2% of the cases in which the patient used Colyte^®^ and MoviPrep^®^, respectively (p<0.001). Another statistically significant difference between the two groups was the percentage of patients using opioids as an outpatient, with 23.1% of the Colyte^®^ group using opioid medications, while only 19.7% of the MoviPrep^®^ group were using opioid medications (p = 0.02). No significant differences were seen between the two groups with respect to age, mean number of polyps, mean size of the largest polyp, gender, requirement for an interpreter, or a chronic pain diagnosis. Additional data on the relationships between covariates and the outcomes from the multivariable analyses are shown in Table 2.

**Table 1:** Patient characteristics by use of Colyte^®^ and MoviPrep^®^.

	Colyte^®^ (n = 1337)	MoviPrep^®^ (n = 1841)	p-value
Age (± S.D.), y	58.8 ± 11.2	58.1 ± 11.8	0.06
Mean Number of Polyps (± SD)	1.1 ± 1.7	1.2 ± 1.8	0.19
Mean Size of the Largest Polyp (± SD), mm	6.3 ± 7.8	7.0 ± 6.4	0.07
Gender (% Women)	54.8	53.1	0.35
Indication (% Diagnostic)	21.5	25.3	0.02
Fellow Involvement (%)	22.7	12.2	<0.001
Required Interpreter (%)	4.2	3.5	0.30
Chronic Pain Diagnosis (%)	24.6	25.4	0.61
Opioid Use (%)	23.1	19.7	0.02
Insurance			<0.001
Commercial (%)	37.2	45.9	
Medicare (%)	34.3	30.3	
Tricare (%)	20.1	19.8	
Medicaid (%)	8.3	4.0	

**Table 2:** Association of covariates with outcomes in multivariable analysis.

**Suboptimal Prep Quality**	Odds Ratio	95% CI	p-value
Age (per year)	1.01	(1.00–1.03)	0.087
Male Gender	1.00	(0.80–1.26)	0.968
Diagnostic Indication	0.90	(0.68–1.20)	0.480
Fellow Participation	0.98	(0.71–1.34)	0.880
Interpreter Needed	0.47	(0.24–0.93)	0.030
Chronic Pain Diagnosis	1.63	(1.26–2.11)	<0.001
Opioid Use	0.93	(0.70–1.25)	0.637
**Adenoma Detection Rate**	Odds Ratio	95% CI	p-value
Age (per year)	1.05	(1.03–1.06)	<0.001
Male Gender	1.26	(1.08–1.48)	0.004
Diagnostic Indication	0.58	(0.47–0.72)	<0.001
Fellow Participation	1.64	(1.30–2.06)	<0.001
Interpreter Needed	1.27	(0.83–1.93)	0.271
Chronic Pain Diagnosis	0.71	(0.58–0.87)	0.001
Opioid Use	1.24	(0.99–1.54)	0.057
**Total Procedure Time**	Coefficient (minutes)	95% CI	p-value
Age (per year)	0.00	(-0.05, 0.05)	0.929
Male Gender	-0.37	(-1.20, 0.47)	0.388
Diagnostic Indication	0.71	(-0.38, 1.79)	0.201
Fellow Participation	8.69	(7.49, 9.90)	<0.001
Interpreter Needed	-5.03	(-7.31, -2.75)	<0.001
Chronic Pain Diagnosis	0.50	(-0.55, 1.55)	0.350
Opioid Use	-0.67	(-1.81, 0.48)	0.253

**Fig 1:**
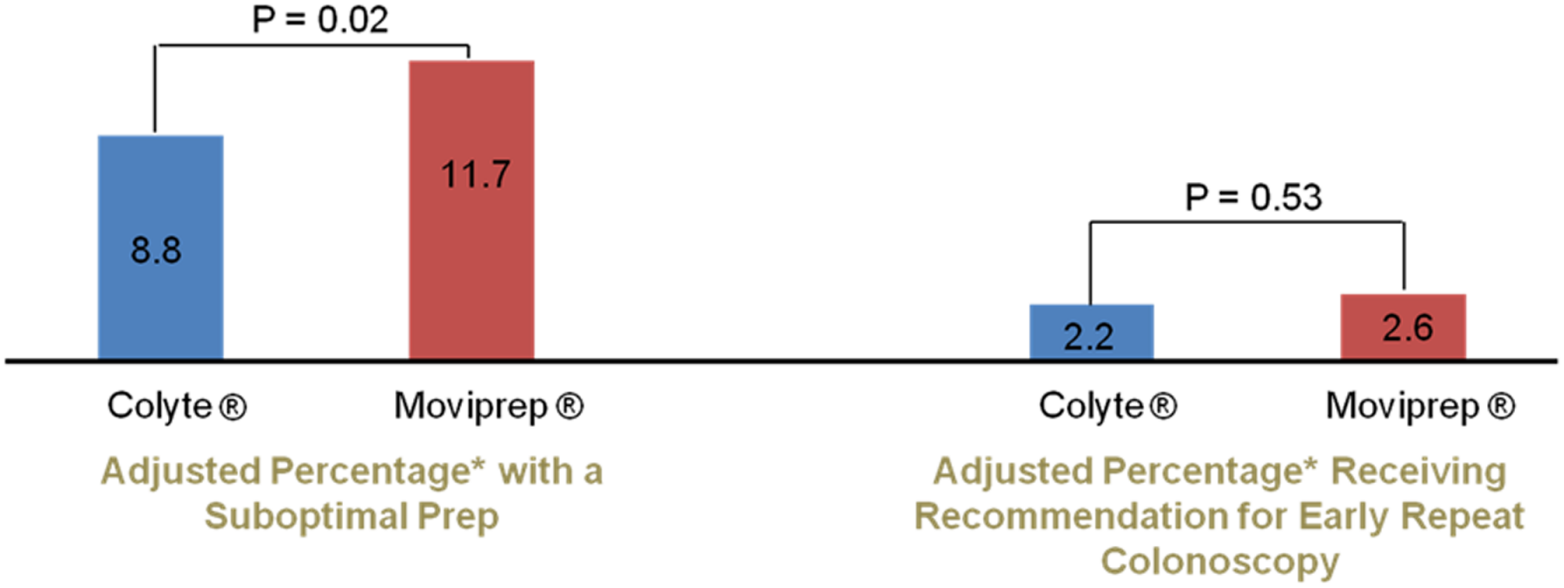
MoviPrep^®^ was associated with a small increase in the odds* of having a suboptimal bowel prep but was not associated with patients receiving a recommendation for an early repeat colonoscopy. *Adjusted for age, gender, fellow participation, the individual endoscopist, insurance provider, chronic pain diagnosis, opioid use, interpreter needed, and whether the colonoscopy was for a diagnostic or screening/surveillance indication.

**Fig 2:**
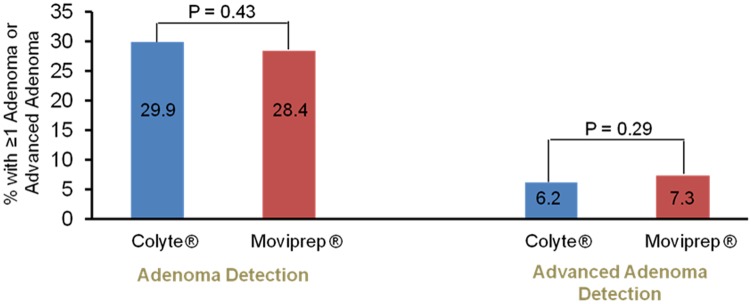
The adenoma detection rate* and the advanced adenoma detection rate* were similar for procedures using Colyte^®^ compared to MoviPrep^®^. *Adjusted for age, gender, fellow participation, the individual endoscopist, insurance provider, chronic pain diagnosis, opioid use, interpreter needed, and whether the colonoscopy was for a diagnostic or screening/surveillance indication.

MoviPrep^®^ was associated with an increase in having a suboptimal bowel preparation (OR 1.36; 95% CI: 1.06–1.76). However, the absolute difference in the adjusted percentage of patients receiving a suboptimal bowel preparation rating between the two bowel preparations was small, with 11.7% of MoviPrep^®^ users and 8.8% of Colyte^®^ users receiving a suboptimal bowel preparation rating (Fig 1). Perhaps more importantly, use of MoviPrep^®^ was not associated with an increase in the odds of receiving a recommendation for an early repeat colonoscopy because of a suboptimal preparation (OR 1.16; 95% CI: 0.72–1.88). The adjusted percentage of patients receiving a recommendation for an early repeat colonoscopy because of a suboptimal bowel preparation was 2.6% for patients who used MoviPrep^®^ and 2.2% for patients who used Colyte^®^. (Fig 1). Patients who used MoviPrep^®^ had a similar ADR (OR 0.93; 95% CI: 0.78–1.11) compared to patients who used Colyte^®^. The adjusted percentage of patients with at least one adenoma was 28.4% in the MoviPrep^®^ cohort and 29.9% in the Colyte^®^ cohort (Fig 2). Similarly, patients who used MoviPrep^®^ had a similar AADR (OR 1.18; 95% CI: 0.87–1.62) compared to patients who used Colyte^®^. The adjusted percentage of patients with at least one advanced adenoma was 7.3% in the MoviPrep^®^ cohort and 6.2% in the Colyte^®^ cohort (Fig 2).

**Fig 3:**
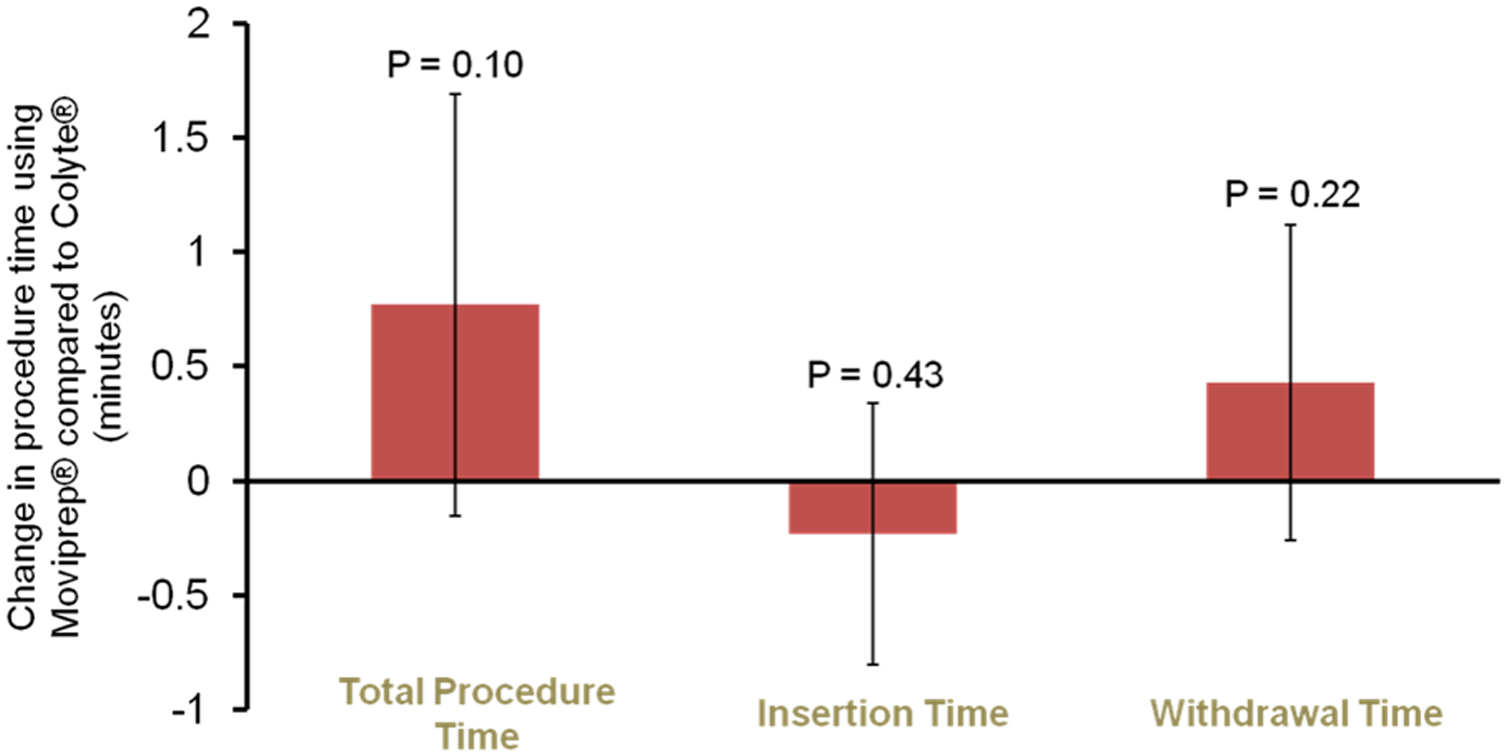
Total procedure times*, insertion times*, and withdrawal times* were similar for procedures using MoviPrep^®^ compared to Colyte^®^. *Adjusted for age, gender, fellow participation, the individual endoscopist, insurance provider, chronic pain diagnosis, opioid use, interpreter needed, total number of polyps, the size of the largest polyp, and whether the colonoscopy was for a diagnostic or screening/surveillance indication. Error bars reflect 95% confidence intervals.

There were not any significant differences in adjusted insertion time, withdrawal time, or total procedure time between the two bowel preparations (Fig 3). The adjusted mean total procedure time was 46.2 seconds longer with MoviPrep^®^ (p = 0.10). The adjusted mean insertion time was 13.9 seconds shorter with MoviPrep^®^ (p = 0.43). The adjusted mean withdrawal time was 25.8 seconds longer with MoviPrep^®^ (p = 0.22).

## Discussion

While other screening measures are available, colonoscopy is the most frequently utilized method for CRC screening [2]. However, the overall CRC screening rate and the effectiveness of colonoscopy at reducing the incidence and mortality from CRC can be further optimized. One patient-reported barrier to colonoscopy is fear of the bowel preparation [15]. Yet, the effectiveness of colonoscopy at reducing an individual’s future incidence and mortality from CRC is dependent upon the quality of the bowel preparation. Low-volume preparations have been rated as superior to high-volume preparations with respect to patient satisfaction and adverse effects like nausea, vomiting, and abdominal pain [6, 10, 11]. Low-volume preparations have become increasingly utilized because of their better patient tolerability, which may reduce fear of the preparation as a potential barrier to colonoscopy. Our study is novel in that it examines the effect of low-volume MoviPrep^®^ against the high-volume Colyte^®^ with respect to unique outcomes, including procedure time characteristics, the recommendation for an early repeat colonoscopy, the ADR, and the AADR.

Our results demonstrate that use of MoviPrep^®^ was associated with a small increase in the odds of having a suboptimal bowel preparation, but this did not translate into any differences in insertion time, withdrawal time, or total procedure time. The absolute difference in suboptimal bowel preparation rates (11.7% for MoviPrep^®^ compared to 8.8% for Colyte^®^) is quite small, and the number who would need to use Colyte^®^ instead of MoviPrep^®^ to prevent one additional suboptimal bowel preparation is 35. More importantly, patients who underwent a colonoscopy using MoviPrep^®^ were not any more likely to receive a recommendation for their next screening or surveillance colonoscopy that was shorter than would otherwise have been clinically indicated compared to those who used Colyte^®^. Although not measured directly, the amount of time endoscopists spent cleaning, irrigating, and aspirating residual stool would have been similar.

Furthermore, the ADR and AADR were similar between those who used MoviPrep^®^ and those who used Colyte^®^, suggesting that similar degrees of visualization were ultimately achieved during those similar procedure times. Since the ADR has been linked as an important measure of colonoscopy’s ability to decrease the future incidence and mortality from CRC [16], the finding that the ADR and AADR were quite similar between the two groups is certainly reassuring. While prior studies have shown that inadequate bowel preparations have led to a decreased ADR, it is not clear how the small increase in the rate of suboptimal bowel preparations in the MoviPrep^®^ group in this study affects their likelihood of developing an interval colorectal cancer [17, 18]. With no difference in adenoma detection rates, it would not appear that the small absolute increase in suboptimal bowel preparation rates for MoviPrep^®^ has much, if any, clinical significance.

There are some limitations to this study. First, this was a cohort study and patients were not randomized to a specific preparation solution. We attempted to account for all potential confounders related to the patients (including gender, age, insurance provider, need for an interpreter, a chronic pain diagnosis, and outpatient use of opiate medications), as well as accounting for the individual endoscopist. While it is possible that other unmeasured variables might change our findings, this does not seem likely. It is possible that the adverse effects of high-volume preparations may drive some patients to not complete the full preparation solution, effectively yielding a low-volume preparation in this subgroup. This might cause the groups to be more similar than one might theoretically postulate, but this may represent a more realistic outcome.

One additional outcome not analyzed in this study is the rate at which patients canceled, re-scheduled, or did not come on the morning of their colonoscopies due to an inability to tolerate the preparation. This subset of patients was not quantifiable for this study. The net result of this would be similar to a patient who presents with an inadequate bowel preparation and then receives a recommendation for an early repeat colonoscopy. This phenomenon would be more likely with a high-volume preparation and the results of including those patients would be more likely to favor low-volume preparations. This is a limitation of this study and one inherent to the cohort design. In the future, a prospective randomized controlled trial could capture bowel preparation failures that lead to colonoscopy appointments being missed or cancelled on the day of the procedure.

One implication of this study is the cost to health insurance carriers, including Medicare and Medicaid, of the bowel preparation in relation to what effect it may have on receiving a recommendation for an early repeat colonoscopy because of an inadequate preparation. The low-volume preparation examined in this study, MoviPrep^®^, is frequently listed as a specialty tier drug without gap coverage amongst Medicare part D plans [19], which may prevent some of those patients from utilizing this better-tolerated prep. If lower-volume solutions are shown to increase adherence to getting a screening colonoscopy, are better tolerated, and ultimately provide an equally effective preparation, then better coverage for these solutions would be justified.

## Conclusions

The low-volume preparation evaluated in this study was associated with a small increase in having a suboptimal bowel preparation, but this difference did not translate into any clinically meaningful differences with respect to recommendations for an early repeat colonoscopy, procedure times, or adenoma detection rates. Mechanisms to reduce financial barriers limiting low-volume preparations should be considered because of their favorable tolerability profile.

## Abbreviations

AADR: advanced adenoma prevalence rate
ADR: Adenoma prevalence rate
CRC: colorectal cancer
PEG: polyethylene glycol
